# Towards a Consensus on Alzheimer’s Disease Comorbidity?

**DOI:** 10.3390/jcm10194360

**Published:** 2021-09-24

**Authors:** Iska Avitan, Yudit Halperin, Trishna Saha, Naamah Bloch, Dana Atrahimovich, Baruh Polis, Abraham O. Samson, Ori Braitbard

**Affiliations:** 1 Bioinformatics Department, Jerusalem College of Technology, Jerusalem 9548311, Israel; iskavitan@gmail.com (I.A.); yudithalperin@gmail.com (Y.H.); 2 Azrieli Faculty of Medicine, Bar Ilan University, Safed 1311502, Israel; trishna.saha27@gmail.com (T.S.); naamah.bloch@biu.ac.il (N.B.); baruhpolis@gmail.com (B.P.); Avraham.Samson@biu.ac.il (A.O.S.); 3 MIGAL, Galilee Research Institute, Kiryat Shmona 11016, Israel; dana.atr@gmail.com; 4 School of Medicine, Yale University, New Haven, CT 06520, USA

**Keywords:** Alzheimer’s disease, oxidative stress, bioinformatics, comorbidity

## Abstract

Alzheimer’s disease (AD) is often comorbid with other pathologies. First, we review shortly the diseases most associated with AD in the clinic. Then we query PubMed citations for the co-occurrence of AD with other diseases, using a list of 400 common pathologies. Significantly, AD is found to be associated with schizophrenia and psychosis, sleep insomnia and apnea, type 2 diabetes, atherosclerosis, hypertension, cardiovascular diseases, obesity, fibrillation, osteoporosis, arthritis, glaucoma, metabolic syndrome, pain, herpes, HIV, alcoholism, heart failure, migraine, pneumonia, dyslipidemia, COPD and asthma, hearing loss, and tobacco smoking. Trivially, AD is also found to be associated with several neurodegenerative diseases, which are disregarded. Notably, our predicted results are consistent with the previously published clinical data and correlate nicely with individual publications. Our results emphasize risk factors and promulgate diseases often associated with AD. Interestingly, the comorbid diseases are often degenerative diseases exacerbated by reactive oxygen species, thus underlining the potential role of antioxidants in the treatment of AD and comorbid diseases.

## 1. Introduction

Alzheimer’s disease (AD) is a chronic neurodegenerative disease that gradually worsens over time [1]. AD is more common among the elderly population, and it is the cause of 60–70% of cases of dementia [2]. Early symptoms of AD include difficulty in remembering recent events, and gradually, bodily functions are lost, ultimately leading to death [3]. The cause of AD is poorly understood, and some of the risk factors are believed to be inherited, involving multiple genes [4]. Recently, we have provided new perspectives on AD as a brain expression of complex metabolic disorders [5]. Other risk factors include oxidative stress, head injuries, depression, obesity, diabetes, hypertension, and Alzheimer’s patients often suffer from other comorbid diseases, as has been reported in recent clinical studies [6,7,8,9]. Treating the comorbid conditions often ameliorates AD symptoms. This trend is cleverly illustrated in an editorial that credits comorbid disease treatment for the decreased prevalence of AD over the past decades [10]. If this trend is to continue, it is key to identify all diseases associated with AD.

Oxidative stress plays a key role in AD [11]. In fact, transient episodes of hypoxia, depriving oxygen from the brain, contribute to the development of AD [12]. Moreover, hypoperfusion associated with oxidative stress is recognized as a common vascular component of AD [13]. Remarkably, oxidative stress also leads to several of the comorbid diseases of AD, such as diabetes [14], hypertension [15], and metabolic syndromes [16]. Antioxidants, quenching reactive oxygen species, have been shown to reverse cognitive deficits in AD [17]. Remarkably, antioxidants have also been shown to provide protection against several comorbid diseases [18]. In the past, we have reported the benefits of several antioxidants in the treatment of AD [19], diabetes [20], and atherosclerosis [21]. Thus, antioxidants present an enormous potential role in treatment of AD and its correlated pathologies. 

Bioinformatics uses the wealth of information to analyze biological data. Text mining and citation counts are often used to identify trends and patterns in medicine [22]. They are powerful technologies for quickly distilling key information from vast quantities of the biomedical literature [23]. Several studies have used text mining, and notably Bork et al. captured the phenotypic effects of a drug based on the side-effects resources published by the FDA [24]. In another study, Jensen and coworkers used text mining to associate diseases and genes, and to establish a web-based database named DISEASE [25]. In the past, we have used frequency analysis of PubMed citations and have shown that antibiotic resistance is periodic [26]. Currently, some 50 million people worldwide are affected with AD, and more than 175,000 PubMed citations are related to Alzheimer’s disease, thus providing a wealth of information [27]. 

We mentioned above that the elderly are at a higher risk of developing dementia. Though, they are also more likely to suffer from concurrent illnesses and comorbidities, which further complicates the diagnosis and sophisticates healthcare. Moreover, AD-associated comorbidity’s economic and medical burden increases substantially with age, making this problem of primary social importance.

Clinicians face dementia-associated comorbidities in daily practice; however, interventional trials do not adequately address their relevance for AD. Apparently, the single-disease therapeutic approach makes treatment strategy less generalizable to most elderly patients with dementia showing multimorbidity. Consequently, the analysis of AD comorbidity patterns is vital for designing efficient therapies.

Here, we present a study that explores the associations between AD and comorbidities and clarifies the shared etiology. Of importance, just a few studies have focused on AD medical comorbidities. A vast majority of them were hospital-based, with no systematic approach. Here, we provide a comprehensive analysis of comorbidity associated with dementia.

We use PubMed citations to document the co-occurrence of Alzheimer’s disease with other pathologies and genes. Then, we cluster the comorbid diseases associated with AD, and provide a visual representation of the disease classification.

## 2. Methods

*List of diseases*. To prepare a testing dataset, we prepared manually curated lists of the most common human diseases. The lists were generated using several medical and biological websites. Then, the lists were merged, and duplicates were removed. Manual editing was performed by iterating duplicate diseases, and finding the most common term in which they are mentioned in the scientific literature as indexed by PubMed. For instance, “Tourette’s syndrome” was replaced with the more widespread term, “Tourette”, “cerebrovascular accident” with “stroke”, “coronary artery disease” with “atherosclerosis”, and so on. We also removed terms associated with common English words, such as “cold”; authors’ name, such as Kawasaki; and University names, such as Marburg. In addition, we replaced multi-word terms describing various symptoms with a single-word term. For example, collective terms used to describe the same disease, such as HIV and AIDS were replaced with the single-word term “HIV”. Finally, to be more specific, the lists were spliced for neurodegenerative diseases, and included several clinical forms of dementia in the general population, such as dementia with Lewy bodies, frontotemporal dementia, and vascular dementia [28]. This splicing was also applied in the case of other general disorders, such as cancer, and specific cancers were added, such as brain cancer, breast cancer, lung cancer, etc.

*PubMed count and disease association.* To mine for disease/disease associations, we queried the PubMed for disease pairs using a Python script with the Wget command. First, we counted the number of PubMed citations returned for the disease pairs (e.g., “Alzheimer” AND “Schizophrenia”). Then, to normalize the association, we divided this number, by the number of citations of each of the diseases alone (e.g., “Alzheimer” OR “Schizophrenia”). The normalization took into account the PubMed abundance of more common diseases, in comparison with the underrepresented rare ones. Finally, the normalized association was multiplied by 100, to obtain a percentage value. The normalized PubMed disease association corresponded to the generalized formula:

Disease Association = [Citations_Disease1+Disease2_]/([Citations_Disease1_] + [Citations_Disease2_]) ∗ 100

*List of genes.* A list of 20,000 human gene symbols and full gene names was assembled from the NCBI, BioMart, and HGCN.

*PubMed count and gene association.* To mine for gene/disease associations, we used PubMed, using a Python script with the Wget command. First, we counted the number of citations returned for a gene and a disease (e.g., “Alzheimer” AND “APP”). Then, to normalize the association, we divided this number by the number of citations returned for each of the terms alone (e.g., “Alzheimer” OR “*APP*”). The normalization took into account the PubMed abundance of more common diseases and genes, compared to the dearth of rare ones. Finally, the normalized association was multiplied by 100, to obtain a percentage value. The normalized PubMed gene–disease association corresponded to the generalized formula:

Gene Association = [Citations_Gene+Disease_]/([Citations_Gene_] + [Citations_Disease_]) ∗ 100

To obtain accurate results for multi-word terms, double quotes were used in all our searches. Our algorithm performed ~1600 searches per hour, and printed the normalized associations to a text file, that could be imported Excel for easy sorting. 

*PCA and cluster analysis.* To cluster diseases associated with Alzheimer’s disease, we used ClustVis [29]. The technique uses principal component analysis (PCA) and provides useful visualization of disease and gene clusters.

## 3. Results

**Figure 1:**
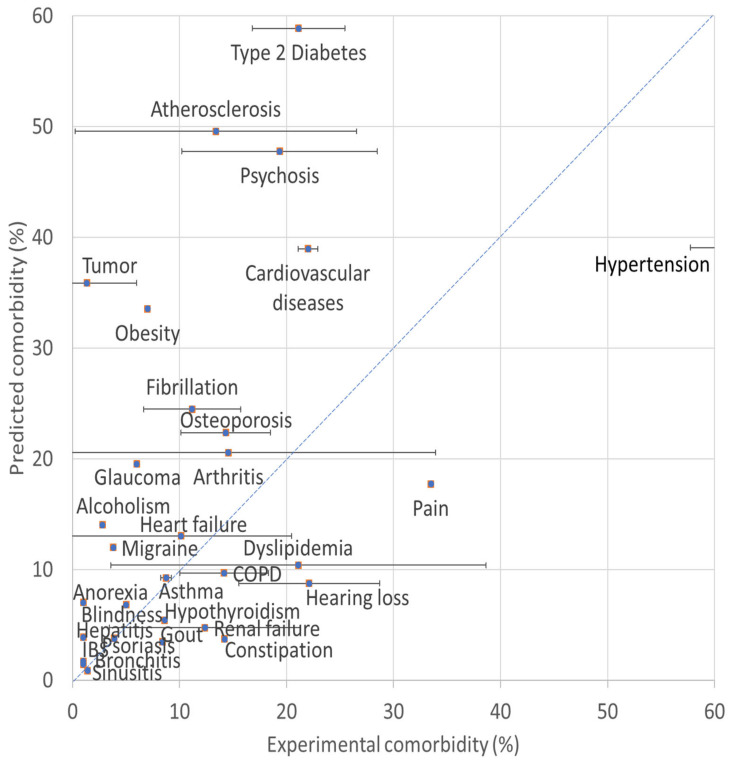
Predicted vs. experimental comorbidity with Alzheimer’s disease. Shown is a plot of the predicted comorbidity association against the experimental average weighted clinical comorbidities of AD. The predicted comorbidity is drawn here as the PubMed normalized disease association multiplied by 100. Notably, the predicted and clinical comorbidities correlate well (R = 0.75). The blue diagonal is a hypothetical match.

*PubMed comorbidity with AD.* Notably, our normalized PubMed associations provide similar Alzheimer’s disease comorbidities as those reported clinically. For example, the 30 non-trivial diseases (and PubMed disease association values) are listed henceforth: schizophrenia (1.04), sleep (i.e., insomnia) (0.66), type 2 diabetes (0.58), atherosclerosis (0.49), psychosis (0.47), hypertension (0.39), cardiovascular diseases (0.38), tumor (0.35), obesity (0.33), fibrillation (0.24), osteoporosis (0.22), arthritis (0.20), glaucoma (0.19), metabolic syndrome (0.19), pain (0.17), herpes (0.15), HIV (0.14), alcoholism (0.14), heart failure (0.13), migraine (0.12), brain tumors (0.11), pneumonia (0.10), dyslipidemia (0.10), COPD (0.09), asthma (0.09), breast cancer (0.09), post-traumatic stress (0.09), hearing loss (0.08), retinopathy (0.07), and anorexia (0.07). Association values below a threshold of 0.07 are considered insignificant and are listed elsewhere (Supplementary Materials Table S1). The 0.07 threshold was selected for several arbitrary reasons: (1) selecting the top 50 results, and (2) discarding results with PubMed co-citation below ~200, and unreliable signal-to-noise ratio (<15). Figure 1 plots the normalized PubMed associations against experimental average clinical comorbidities of AD. The correlation between the predicted comorbidity (listed in Supplementary Materials Table S1) and the experimental comorbidity (listed separately in Table 1) is notable (R = 0.75). Remarkably, the correlation is even higher with each of the experimental reports separately, with Leese et al. (R = 0.93) in the lead [30], followed by Wang et al. [8] (R = 0.82), Tortajada et al. [6] (R = 0.81), Browne et al. [7] (R = 0.79), and Yen et al. [9] (R = 0.79). Interestingly, our citation counts show that Alzheimer’s disease is also associated with pathologies prevalent at younger age, and not only with neurodegenerative old-age disorders. As such, our findings are useful for illustrating the most important risk factors and comorbidities of AD. 

For example, in agreement with our findings, schizophrenia (1.04) and psychosis (0.47) are relatively common side effects of AD [31]. Likewise, sleep (0.66)—in particular, lack thereof (<7 h/night)—has recently been correlated with dementias [32]. Type 2 diabetes (0.58) is a comorbid disease strongly associated with AD [33], and to a much higher degree than type 1 diabetes (0.05). Moreover, atherosclerosis (0.49) ranks high on the list of diseases associated with AD, and it is becoming a more widely recognized biomarker of AD [31]. In Figure 1, type 2 diabetes and atherosclerosis deviate significantly from the diagonal, and their predicted comorbidity is much higher than that experienced clinically. Figure 1 indicates that type 2 diabetes and atherosclerosis may be significantly underdiagnosed in the clinic, this potentially aggravating AD if left untreated. Next, hypertension (0.39) is a major risk factor for AD, and has been associated with an increased risk of up to 25% in developing AD [34]. Likewise, growing evidence supports a strong and likely causal association between cognitive decline and Alzheimer’s disease with incidence of cardiovascular disease (0.38), such as heart failure (0.13), and its risk factors, hypertension (0.39), atherosclerosis (0.49), and fibrillations (0.24) [35]. Interestingly, our data suggest that atherosclerosis alone, is more likely to contribute towards AD, than cardiovascular diseases (e.g., atrial fibrillation, tachycardia, etc.) without it. Similarly, the metabolic syndrome (0.19), which combines diabetes (0.58), obesity (0.33), hypertension (0.39), and dyslipidemia (0.10), is strongly associated with AD. Pain (0.17) and migraine (0.12) are also associated with AD [36], and often treating comorbid diseases alleviates AD symptoms, such as pain [37] and depression [38]. The major pain (0.17) associated with AD is joint pain (0.15), such as arthritis (0.20) and headaches (0.22), such as migraines (0.12), but not with skin pain (0.00); not with stomach pain (0.00), such as ulcers (0.03); not with mouth pain (0.00), such as caries (0.02); and not with back pain (0.06). Finally, lung diseases, and in particular COPD (0.09) [39], and asthma (0.09) have been associated with AD [39].

Our disease association also highlights disorders with higher incidence among women. Compared to men, women suffer more from AD, osteoporosis, arthritis, and migraines. Osteoporosis (0.22) and AD are both degenerative diseases, which are often comorbid in elderly women [40]. Likewise, arthritis (0.20) is an autoimmune disease which increases the risk of developing cognitive impairment [41], as well as other disease associated with old age [42]. Interestingly, women (22.5%), are twice as likely as men (11.9%) to suffer from migraines (0.12) associated with metabolic syndrome (0.19) [43].

Cancer and AD are both associated with aging, yet they do not often coincide. In fact, AD and cancer are inversely related [44], as shown by our low PubMed association values. For example, lung cancer (0.04), colorectal cancer (0.03), pancreatic cancer (0.01), bladder cancer (0.01), prostate cancer (0.06), skin cancer (0.01), and kidney cancer (0.004) are unlikely to develop in AD patients. Interestingly, the association with breast cancer (0.09) and tumor (0.35) is significant, as women treated with chemotherapy are more prone to develop AD, compared to their untreated counterpart [45]. Moreover, Figure 1 suggests that tumor deviates significantly from the diagonal. This point illustrates the absence of causality in our study, namely that chemotherapy results in AD, but AD does not result in breast cancer. Likewise, prostate cancer is associated with AD only following androgen deprivation therapy for prostate cancer. Interestingly, brain tumors (0.11) are associated with distinct “pseudo AD”, as cognitive decline after brain cancer treatment are pronounced [46]. The AD–tumors citation metric is 0.35, while those for AD–individual cancers are all below 0.11. Notably, the AD–tumors citation metric (0.35), is close to the sum of the AD–individual cancers [lung cancer (0.04) + colorectal cancer (0.03) + pancreatic cancer (0.01) + bladder cancer (0.01) + prostate cancer (0.06) + skin cancer (0.01) + breast cancer (0.09) + brain tumors (0.11) kidney cancer (0.004) = 0.35]. Thus, the AD–individual cancers listed here could contribute to the vast majority of all AD–tumor associations. AD and cancer are inversely related, yet together they rank among the leading causes for human death around the world. AD and cancer are the response to mutations (sometimes acquired from reactive oxygen species), in the presence of an unbalanced immune system. Balance is key, and immune modulation (also through antioxidants) is a sweet-spot between these two lethal outcomes [47].

Complaints related to poor eyesight, and even blindness (0.06), are common in AD patients. Usually, the poor eyesight is due to retinopathy (0.07) associated with glaucoma (0.19) which is form of neurodegenerative disease [48]. Similarly, hearing loss (0.08) is often associated with the neurodegeneration in general, and with AD in particular [49]. 

Infectious agents have also been associated with AD. Herpes (0.15) and HIV (0.14) are associated with AD, whereas other viruses, such as influenza (0.05), hepatitis (0.02), rabies (0.01), varicella (0.01), measles (0.01), and dengue (0.009) are not. A link between AD and the herpes simplex virus (HSV) was established over 30 years ago, yet its role remains controversial to date [50]. Since then, researchers have bolstered the association between AD and HSV1, HHV6A, and HHV6B in particular. Interestingly, HIV has been associated with an AD-like neurocognitive disorder, yet a causative link has yet to be identified [51]. In particular, HIV-1 subtype D, which preferentially binds to CXCR4 on T cells, has been linked with HIV-associated neurocognitive disorder. Bacterial and parasitic infections have been associated with AD, yet PubMed associations do not attribute the responsibility of any particular agent. Conditions such as pneumonia (0.10) are also not highly associated with AD, and are likely caused by the difficulty of swallowing in AD patients. 

Heavy drinking, alcoholism (0.14), and smoking tobacco (0.07) accelerate shrinkage or atrophy of the brain, which in turn is a critical determinant of neurodegenerative changes and cognitive decline in AD. More hard liquor is also associated with a faster rate of cognitive decline [52]. The list goes on and includes low PubMed associations, such as cystic fibrosis (0.06) and periodontal (0.06), which are considered insignificant. In summary, we find the associations extremely useful, and they are important in light of the failures to explain the etiology of AD. 

*Trivial associations disregarded.* Trivially, the first 10 most associated diseases with Alzheimer’s are mostly neurodegenerative diseases (and their calculated disease associations) as listed below: dementia (21.16), Parkinson’s disease (6.07), vascular dementia (3.08), Huntington’s disease (2.07), frontotemporal dementia (2.01), depression (1.38), dementia with Lewy bodies (1.37), multiple sclerosis (1.13), Down’s syndrome (1.12), and senile dementia (1.09). Not surprisingly, dementia is listed among the first comorbidities, considering Alzheimer’s disease is the most common type of dementia (60–70%) [28]. Moreover, vascular dementia, frontotemporal dementia, dementia with Lewy bodies, and senile dementia, feature high on this list too, as they are similar neurodegenerative syndromes on the dementia spectrum [53]. Despite many differences, Parkinson’s disease and Huntington disease are also highly co-cited with AD, as both diseases are chronic illnesses of old-age and often overlap in the textbooks of neurodegenerative diseases. These associations are trivial, and are ignored. Furthermore, multiple sclerosis (MS), is greatly co-cited with Alzheimer’s, even though clinical comorbidity is insignificant, and brain regions and cognitive functions differ greatly in patients with AD and patients with MS [54]. The first non-neurodegenerative disease, appearing on the list is depression. As evidenced extensively, depression is among the most frequent psychiatric ailment in dementia, and depressive symptoms are risk factors for AD [55]. Notably, the term depression also refers to a systematic decline, a financial recession, or a hollow impression, and multiple citations refer to meanings other than psychiatric depression. Finally, Down’s syndrome, is also highly associated with AD, and trisomy 21 carriers of amyloid precursor protein develop early onset AD [56]. However, these associations are trivial, and may be discarded as insignificant. Likewise, the lower-ranking trivial terms are ignored, and synonyms of neurodegeneration, such as epilepsy (0.71), Creutzfeldt–Jakob (0.55), encephalopathy (0.51), encephalitis (0.41), ataxia (0.32), apraxia (0.21), pre-senile dementia (0.19), neuropathy (0.14), myoclonus (0.13), chorea (0.09), encephalomyelitis (0.08), motor neuron (0.07), and Kuru (0.05), were disregarded as trivial. 

**Figure 2:**
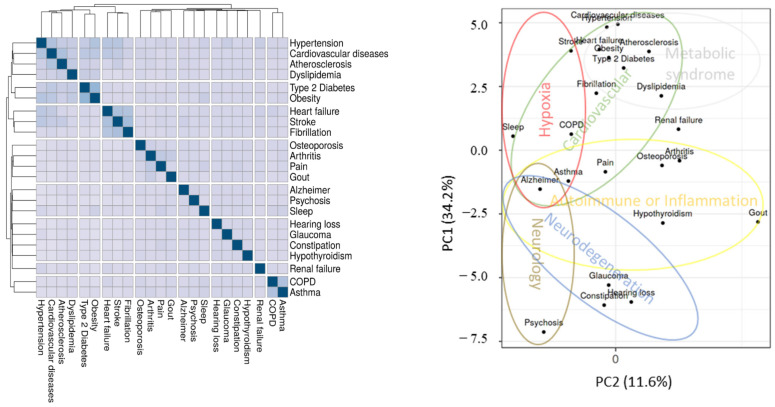
Clustering of diseases closely associated with Alzheimer’s disease (PubMed). Shown on left is a heatmap of the comorbid diseases on PubMed. Background colors indicate association degree, and values vary from 0% (in white) to 100% (in blue). Shown on the right is a principal component analysis of the same association data.

*Clustering of diseases associated with AD.*Figure 2 shows a clustered heatmap based on the association values of a set of diseases highly associated with Alzheimer’s disease (AD). The set of diseases shown in Figure 2 incorporates the main diseases listed earlier, clustered in a heatmap and plotted representatively using PCA. 

Notably, commonly associated risk factors are clustered together, and vice versa. For example, COPD and asthma are closely associated, despite clinical variations. On the other hand, psychosis and hypertension are distantly associated, as they have little in common, except perhaps with AD. The PCA plot illustrates the inter-disease clustering, relative to AD shown in the center left. Coarsely, the hypoxia cluster (red circle) includes AD, sleep apnea, asthma, COPD, and stroke. The cardiovascular cluster (green circle) includes AD, atherosclerosis, dyslipidemia, hypertension, atrial fibrillations, heart failure, and coincides with the metabolic-syndrome cluster (gray circle) encompassing type 2 diabetes, and obesity. The autoimmune/inflammation cluster (yellow circle) includes arthritis, osteoporosis (i.e., Hashimoto’s thyroiditis), asthma, gout, and pain. The neurodegeneration cluster (blue circle) includes AD, hearing loss such as auditory nerve degeneration, glaucoma leading to optic nerve degeneration, and constipation such as enteric system neurodegeneration. The neurology cluster (brown circle) includes AD and psychosis, and is trivial. Renal failure does not cluster readily with other AD comorbidities, and appears orphaned somewhere between cardiovascular diseases, metabolic syndrome, and autoimmune diseases. Importantly, the inter-disease associations of the PCA plot are not to scale, and orthogonal distances are better viewed in the heatmap. As such, AD is a spectrum of neurodegenerative disorders, and combines a range of diseases related mostly to old age. The AD spectrum includes chronic hypoxia [12], cardiovascular insufficiency [36], metabolic syndrome [57], autoimmune diseases, and inflammation [58]. 

*Gene association values also concur with clinical data.* To address our findings from a more biological perspective, we explore the association of genes. Of our 20,000 human genes, the top six associated with AD (and gene association values) are *APP* (6.06), *APOE* (4.83), *PSEN1* (2.83), *APPBP2* (1.94), *BACE1* (1.89), and *MAPT* (1.81). Notably, the amyloid precursor protein (*APP*), presenilin (*PSEN1*), and microtubule-associated protein Tau (*MAPT*), and they are among the top genes used in transgenic mouse models of AD (*APP* KM670/671NL, *MAPT* P301L, and *PSEN1* M146V) [15]. Likewise, apolipoprotein E (*APOE*) and Beta-secretase 1 (*BACE1*) are among the genes used to model AD in mice. These six genes are trivially overrepresented, as they are often used to model AD. Of the 20,000 human genes, the subsequent non-overrepresented 19,994 genes are also interesting, and they are used to genetically associate AD with other diseases.

**Figure 3:**
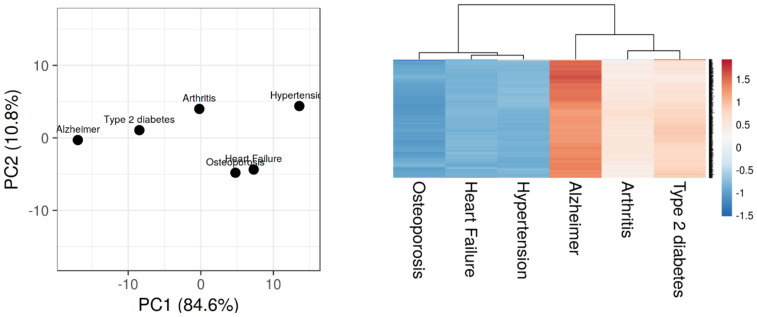
Gene association of Alzheimer’s disease with representative diseases. Shown on the left is PCA clustering of 5 representative diseases associated with AD, according to 20,000 human gene associations on PubMed. Shown on the right is a heatmap of the same 5 diseases showing the top 500 genes associated with AD. The figures were prepared using ClustVis [29]. Red and blue lines indicate high and low gene association, respectively.

To mine for gene/disease associations, we queried the PubMed and counted the number of citations returned for a disease and all 20,000 human genes (e.g., “Alzheimer” AND “*APP*”). Figure 3 shows the PCA clustering and heatmap of five representative diseases associated with AD, using 20,000 human gene association values. In this diagram*,* the closest neighbors of AD are type 2 diabetes, hypertension, and arthritis. The clustering of diseases according to genetic association is in agreement with our ranking of Alzheimer’s disease association (Supplementary Materials Table S1), and diabetes (0.59), hypertension (0.39), and arthritis (0.2) are often comorbid with AD (Table 1). These genetic associations are more significant, as they share more genes associations in PubMed citations. On the other hand, osteoporosis (0.22) and AD are linked less through genes association values, than observed through clinical comorbidity (Table 1). Perhaps, this is because osteoporosis and AD both affect women to a higher degree due to higher longevity and stronger autoimmunity, but not because they share many genes, or similar biological pathways, according to PubMed gene association values. Clearly, AD is phylogenetically isolated from the representative comorbid diseases, and shares less genes than say hypertension and heart failure. This isolation reflects our limited understanding of the genetic etiology of AD. As more genetic testing become available, more clear genetic signatures are expected to ensue.

## 4. Discussion

In this study, we searched for comorbid disorders associated with Alzheimer’s disease. We used PubMed citations to document the co-occurrence of Alzheimer’s disease with other pathologies and genes. Remarkably, our association values correlate with previous clinical research and attest to the strength of data mining. We find AD to be strongly associated with a broad spectrum of severe degenerative disorders, which include chronic hypoxia [12], cardiovascular insufficiency [35], metabolic syndrome [57], autoimmune diseases, and inflammation [17]. While AD therapies are limited, we propose to treat AD-associated diseases with them to attenuate clinical manifestation and increase longevity [10]. Notably, antioxidants have been used to treat comorbidities of AD [17].

Multimorbidity is very common in AD. Schizophrenia, depression, diabetes, osteoporosis, etc., are strongly associated with AD incidence. Accordingly, comorbidity treatment has to be carefully integrated into the current strategies to cure AD patients. It is well known that women are disproportionately affected by AD, which correlates with the current clinical data [8]. Additionally, we show a strong link between schizophrenia and AD. Recent clinical data prove that schizophrenia patients have a significantly higher risk (about three times) of developing AD than general population [58]. Of note, schizophrenia is one of the prevalent psychiatric diseases with a robust genetic component; however, its precise transcriptomic signature remains undetermined. Accordingly, focusing upon the patients who develop dementia may be a promising approach to decipher the etiological heterogeneity of these complex and diverse pathologies, such as AD and schizophrenia.

*Towards a consensus of clinical AD comorbidities.* Here, we refer the reader to five notable previous reports [6,7,9,30,59] reviewed in Table 1. Table 1 summarizes these reports and provides a detailed clinical evaluation of comorbid diseases with AD. The table also provides the calculated weighted average for each comorbid disease, as well as the standard deviation (SD). The number of participants (N) is listed beneath each of the reports. 

In most cases, the comorbid percent values are comparable among different studies. In some cases, however, the comorbidity varies by more than 30%. Notably, the list of comorbidities is partial only, and does not include less common associated diseases. The associated diseases, and their frequency, can also be revealed through our PubMed disease association, as shown earlier.

As an alternative, AD and its related comorbidities could also have been studied from the multiple causes of death data on the US population. This database was used by Yashin and coworkers [60]. In all cases, much care should be taken so as not to overinterpret causality.

*Potential pitfalls.* Text mining of PubMed citations is taken at face value. Although PubMed is expected to contain most of our current knowledge, citation counts do not necessarily reflect this knowledge. PubMed citations could contain much noise, and careful analysis is required before reaching conclusion. Random noise is expected to rise a square-root function of real data, and hundreds of citations are required for statistical significance. Non-random noise must be filtered out logically, using data normalization, and care must be taken so as not to overinterpret the data.

Our search method does not entirely prevent false-positive counts of disease–disease citations. To reduce false-positives, only the title, abstract, and keywords were mined, and not the entire paper. The title, abstract and keywords are a distilled version of the paper, and as such are expected to contain less false-positive contamination. In addition, the false positives are partially prevented, by dividing the co-citations with each of the citations alone. As such, common diseases, which are more likely to give rise to false-positives, are given less importance. Finally, false-positives stemming from random co-citations rise to the squared root power (i.e., random walk) of true positives, and are considered as background noise. The false-positives cannot be completely prevented, unless each co-citation is read carefully, an enormous task which was not undertaken. Notably, our method is a good-enough approximation, as attested by the validation and correlation with experimental data.

Cause or effect? Treating the comorbid conditions often ameliorates AD symptoms. This trend has been illustrated in an editorial that credits comorbid disease treatment for the decreased prevalence of AD over the past decades [10]. However, most sick individuals in developed societies use medications and get medical treatments. In theory, AD and its comorbid diseases could also be caused by the side effects of treatments. The treatment causality has been discussed earlier, in connotation with cancer and AD, where chemotherapy leads to AD. Thus, as unlikely as it may sound, treatment of any comorbid disease could also aggravate AD, and hypothetically speaking any drug against type 2 diabetes may worsen AD. In pharmacology, we often apply the rule “everything can be good, but only in the right measure”. Thus, treating the comorbid condition is good, but only in the right measure.

*Dementia taxonomy.* AD dementia correlates with multiple syndromes related to old age and is characterized by the buildup of misfolded proteins into β-amyloid plaques and tau neurofibrillary tangles. Although this naming convention is ambitious, it may be helpful to differentiate between deposition of β-amyloid and tau proteins. Specificity may help distinguish between various pathologies at early stage in order to treat them appropriately. This specificity would respect other naming conventions, such as Lewy-body dementia, which is defined by the buildup of α-synuclein, and Huntington’s disease, which is defined by the build-up of huntingtin. In addition, non-specific dementia names, such as senile dementia, vascular dementia, frontotemporal dementia, and sub-cortical vascular dementia, should be used only if no protein deposit is observed. However, if protein deposits are found, then the corresponding proteopathy must be named accordingly. The nomenclature of AD should (at least) be as accurate as that used in cancer research, where nobody in their right mind would classify cancer as a single disease. Therefore, the most serious problem in the field that should be urgently addressed is the development of precise diagnostic tools and biomarkers which could detect the disease at very early stage, distinguish between its clinical variants, and be helpful in the treatment efficacy monitoring. Correct use of AD nomenclature is expected to increase our understanding and help the scientific community develop individualized treatments. 

*Antioxidants can alleviate AD and its comorbidities.* Notably, most of the risk factors associated with Alzheimer’s disease are all related to extensive ROS generation and chronic inflammation. These risks include stroke, atrial fibrillation, coronary heart disease, hyperlipidemia, hypertension, sleep deprivation, diabetes mellitus, heart failure, peripheral vascular disease, renal failure, chronic obstructive pulmonary disease, valvular heart disease, tobacco use, and alcohol abuse. Treating oxidative stress and relieving inflammation are promising therapeutic approaches to AD and its comorbidities [17,61]. Nevertheless, antioxidants are potential double-edged swords, and improper modulation of their targets, off-site pharmacodynamic effect, and their potential pro-oxidant effects may cause harm in addition to benefit [62]. In addition, readily available antioxidants suffer from patentability issues and have an extremely low history of FDA approval. While we anticipate the antioxidant Reactome, treatment of AD comorbidities by using FDA-approved drugs remains a high priority. 

## 5. Conclusions

We used data mining and sifted through vast amounts of information on PubMed to discover patterns associated with AD. We found that AD is usually not a detached disease, and it is often associated with other conditions. We classified comorbid diseases into six categories, namely hypoxic, cardiovascular, metabolic syndrome, inflammatory, neurodegenerative, and neurologic diseases. We found that some comorbid diseases may go unnoticed in the clinic (i.e., type 2 diabetes and atherosclerosis), thus potentially aggravating AD.

## Figures and Tables

**Table 1:** Previously reported clinical comorbidities of Alzheimer’s disease, and consensus.

	Clinical Comorbidity (%)					Average Clinical Comorbidity (%)
Report Source	Leese et al. [30]	Yen et al. [9]	Browne et al. [7]	Wang et al. [8]	Tortajada et al. [6]	
Participants (N)	~8331	132,405	4999	2618	100	148,453
Alzheimer	100	100	100	100	100	100
Hypertension	40	72	53.4	55	51	69.3 (12.5) ˟
Pain			33.5			33.5
Dyslipidemia				20.2	45	21.1 (17.5) ˟
Cardiovascular Diseases			21.6	22.8	21	22.0 (0.9) ˟
Type 2 Diabetes	18	21.5	14	25.7	19	21.1 (4.3) ˟
Hearing loss			22.3		13	22.1 (6.6) ˟
Depression	17	20.4	23.5	32.3	27	20.5 (5.9) ˟
Psychosis	4	21.4	1		12	19.3 (9.1) ˟
Arthritis			2.1	38.2	8	14.5 (19.4) ˟
Osteoporosis				14.1	20	14.3 (4.2) ˟
Constipation			14.2			14.2
COPD	8	14.9	6.9	10.8	4	14.2 (4.1) ˟
Atherosclerosis			4.1	22.7		13.4 (13.2) ˟
Stroke	10		17.2			12.7 (5.1) ˟
Renal failure		13.3	20.8	3		12.4 (8.9) ˟
Fibrillation			13.4	7		11.2 (4.5) ˟
Heart failure	14	10.1	6.3	5.8	31	10.1 (10.4)
Asthma	9		8.3			8.7 (0.5) ˟
Hypothyroidism			8.6			8.6
Gout				8.4		8.4
Obesity					7	7.0
Parkinson	8		2.9			6.1 (3.6) ˟
Glaucoma					6	6.0
Blindness			5			5.0
Epilepsy	6		1.9			4.0 (2.9) ˟
Psoriasis			3.9			3.9
Migraine			3.8			3.8
Alcoholism			2.8			2.8
Sinusitis			1.4			1.4
Tumor		1.2		7.8		1.3 (4.7) ˟
Multiple sclerosis			<1			<1
Anorexia			<1			<1
Hepatitis			<1			<1
Irritable bowel syndrome (IBS)			<1			<1
Bronchitis			<1			<1
Cirrhosis			<1	0.5		<0.8 (0.3) ˟

˟ Standard deviation (%).

## Data Availability

Not applicable.

## Supplementary Materials

The following are available online at https://www.mdpi.com/article/10.3390/jcm10194360/s1, Table S1: Disease-Disease association.

## Abbreviations

AD—Alzheimer’s disease; *APP*—amyloid precursor protein; *APOE*—apolipoprotein E; *BACE1*—Beta-secretase 1; COPD—chronic obstructive pulmonary disease; HIV—human immunodeficiency virus. HSV—herpes simplex virus; MS—multiple sclerosis; PCA—principal component analysis; *PSEN1*—presenilin 1; ROS—reactive oxygen species. SD—standard deviation.
