# Supplementary material for: Towards a Consensus on Alzheimer’s Disease Comorbidity?
Source: J Clin Med. 2021 Sep 24;10(19):4360. doi: 10.3390/jcm10194360 (PMC8509357; doi:10.3390/jcm10194360)
Supplement: Supplementary file 1 [file jcm-10-04360-s001.zip › jcm-1308093-supplementary.pdf]

Supplementary Table S1.

Rank	Disease	"Alzheimer" AND "Disease"	"Alzheimer" OR "Disease"	Association (100%)
1	alzheimer	177074	177074	100
2	dementia	55447	261935	21.168229
3	Parkinson	17655	290790	6.0713917
4	vascular dementia	5556	180202	3.0832066
5	huntington	4233	204164	2.0733332
6	frontotemporal dementia	3644	181266	2.0103053
7	Depression	8195	592079	1.3841058
8	dementia with lewy bodies	2461	178960	1.3751676
9	Stroke	6,509	519,046	1.2540314
10	Multiple sclerosis	2977	262667	1.1333742
11	down syndrome	2294	204035	1.1243169
12	senile dementia	1957	178017	1.0993332
13	schizophrenia	3349	321933	1.0402786
14	Diabetes	7,130	915,314	0.7789677
15	Epilepsy	2328	327716	0.7103712
16	sleep	2704	404,608	0.6683012
17	Type 2 diabetes	2159	366745	0.5886924
18	creutzfeldt jakob	1030	184520	0.5582051
19	encephalopathy	1186	231698	0.5118732
20	Atherosclerosis	1591	320967	0.4956896
21	Psychosis	1064	222,741	0.4776848
22	encephalitis	968	231781	0.4176356
23	Hypertension	2769	708660	0.3907374
24	Cardiovascular diseases	1529	392485	0.389569
25	Tumor	7092	1,976,321	0.3588486
26	Obesity	1775	529312	0.335341
27	ataxia	714	222373	0.3210821
28	Fibrillation	732	299,112	0.2447244
29	Osteoporosis	602	269117	0.2236945
30	trisomy	433	198408	0.2182372
31	apraxia	392	180928	0.2166608
32	Arthritis	897	436174	0.2056519
33	Glaucoma	491	251242	0.1954291
34	metabolic syndrome	452	237300	0.1904762
35	Pain	1752	988,516	0.1772354
36	presenile dementia	300	177360	0.1691475
37	scrapie	307	182223	0.1684749

38	herpes	387	248666	0.1556304
39	hiv	818	551155	0.1484156
40	neuropathy	373	254293	0.1466812
41	Alcoholism	373	265818	0.1403216
42	myoclonus	253	187623	0.1348449
43	watkins	250	190750	0.1310616
44	Heart failure	518	397412	0.1303433
45	Migraine	261	217346	0.120085
46	brain tumors	235	201741	0.116486
47	pneumonia	397	377297	0.1052221
48	Dyslipidemia	219	210858	0.1038614
49	COPD	268	276781	0.0968275
50	breast cancer	445	469932	0.0946946
51	chorea	174	184878	0.0941161
52	Asthma	345	372746	0.0925563
53	post traumatic stress	198	217497	0.0910357
54	Hearing loss	220	251,264	0.0875573
55	encephalomyelitis	165	203147	0.081222
56	cold	272	344009	0.0790677
57	motor neurone	136	179216	0.0758861
58	kawasaki	162	214366	0.0755717
59	retinopathy	172	233105	0.0737865
60	marburg	149	205676	0.072444
61	Anorexia	149	212318	0.0701778
62	periodontal	180	260671	0.0690526
63	Blindness	153	224,775	0.0680681
64	coronavirus	168	259679	0.0646953
65	cystic fibrosis	147	230996	0.0636375
66	osteoarthritis	173	272491	0.0634883
67	allergy	246	394937	0.0622884
68	sars	139	225781	0.0615641
69	kuru	105	178659	0.0587712
70	sepsis	181	308999	0.0585762
71	narcolepsy	102	182708	0.0558268
72	myasthenia gravis	109	195471	0.0557627
73	Hypothyroidism	120	220873	0.0543299
74	leukemia	263	494767	0.0531563
75	diarrhea	146	283229	0.0515484
76	muscular dystrophy	104	203650	0.051068
77	myopathy	101	198849	0.0507923
78	malaria	140	276775	0.0505826
79	hypothermia	113	223745	0.0505039
80	type 1 diabetes	135	267618	0.050445

81	Influenza	147	294522	0.0499114
82	meningitis	119	243902	0.0487901
83	Cirrhosis	151	311308	0.048505
84	Renal failure	131	275539	0.0475432
85	chlamydia	98	207503	0.0472282
86	lymphoma	190	403304	0.0471109
87	dehydration	99	216299	0.04577
88	lupus erythematosus	116	253721	0.0457195
89	cardiomyopathy	113	260095	0.0434457
90	lung cancer	149	346774	0.0429675
91	melanoma	135	316878	0.0426031
92	neoplasm	378	920590	0.0410606
93	colorectal cancer	111	286019	0.0388086
94	Hepatitis	173	446,905	0.0387107
95	pertussis	79	207732	0.0380298
96	Psoriasis	86	227773	0.0377569
97	chagas	76	202907	0.0374556
98	Constipation	77	206720	0.0372485
99	graves	77	209267	0.0367951
100	Gout	68	196260	0.0346479
101	ulcers	76	238798	0.0318261
102	siderosis	57	179371	0.0317777
103	cerebral palsy	65	205787	0.0315861
104	colitis	80	259847	0.0307873
105	retinitis	60	194898	0.0307853
106	tourette syndrome	50	182457	0.0274037
107	infertility	74	274217	0.0269859
108	tetanus	55	206421	0.0266446
109	fibromyalgia	49	189317	0.0258825
110	cholera	53	205990	0.0257294
111	hepatitis c	69	271096	0.0254522
112	iron deficiency	51	202885	0.0251374
113	tuberculosis	109	442510	0.0246322
114	nephritis	52	211490	0.0245875
115	angina	58	248289	0.0233599
116	hodgkin	59	256852	0.0229704
117	tinnitus	43	191103	0.022501
118	adrenoleukodystrophy	40	179499	0.0222842
119	erectile dysfunction	45	202407	0.0222324
120	myeloma	53	243463	0.0217692
121	ulcerative colitis	49	227939	0.021497
122	chronic fatigue syndrome	37	184202	0.0200866
123	pancreatic cancer	42	219507	0.0191338

124	progeria	34	179260	0.0189669
125	lead poisoning	36	189923	0.0189551
126	dermatitis	51	272513	0.0187147
127	nervous colon	34	182267	0.018654
128	cushing	36	197328	0.0182437
129	caries	42	239945	0.017504
130	syphilis	37	212575	0.0174056
131	liver cancer	35	202418	0.017291
132	Irritable bowel syndrome	33	192097	0.0171788
133	purpura	35	211814	0.0165239
134	lyme	31	191966	0.0161487
135	rabies	31	193331	0.0160347
136	hyperthyroidism	34	212228	0.0160205
137	paget	30	187890	0.0159668
138	rheumatism	31	194959	0.0159008
139	weil	28	186205	0.0150372
140	bronchitis	31	210231	0.0147457
141	Bronchitis	31	213,624	0.0145115
142	hypotonia	27	187487	0.014401
143	plague	27	188229	0.0143442
144	emphysema	29	212542	0.0136444
145	sickle cell anemia	27	200152	0.0134897
146	salmonella	36	272213	0.0132249
147	diphtheria	26	198198	0.0131182
148	leprosy	27	205950	0.01311
149	mad cow	21	177412	0.0118369
150	bladder cancer	25	212969	0.0117388
151	acne	23	196338	0.0117145
152	osteomalacia	21	184190	0.0114013
153	uremia	22	199840	0.0110088
154	rhinitis	24	223256	0.01075
155	hepatitis b	30	279532	0.0107322
156	economo	19	177374	0.0107118
157	gastritis	22	207750	0.0105897
158	avitaminosis	19	180088	0.0105504
159	varicella	20	192058	0.0104135
160	myopia	20	202095	0.0098963
161	measles	20	205848	0.0097159
162	dengue	19	201880	0.0094115
163	glomerulonephritis	21	223869	0.0093805
164	pulmonary embolism	21	231939	0.0090541
165	Sinusitis	18	205071	0.0087774
166	bulimia	16	188030	0.0085093

167	spina bifida	15	186107	0.0080599
168	jaundice	17	220456	0.0077113
169	abscess	19	261022	0.0072791
170	gerd	11	187416	0.0058693
171	trypanosomiasis	11	189656	0.0058
172	phenylketonuria	10	183070	0.0054624
173	myelitis	10	183986	0.0054352
174	mucous	11	220375	0.0049915
175	gingivitis	9	191294	0.0047048
176	enteritis	9	193300	0.004656
177	peritonitis	10	218200	0.004583
178	kidney cancer	8	182785	0.0043767
179	coeliac	8	185462	0.0043136
180	shingles	6	178589	0.0033597
181	sids	6	181121	0.0033127
182	icterus	5	180002	0.0027777
183	common cold	5	183530	0.0027244
184	thalassaemia	4	182033	0.0021974
185	histiocytosis	4	190400	0.0021008
186	tooth decay	3	178445	0.0016812
187	laryngitis	3	181229	0.0016554
188	septicaemia	3	183543	0.0016345
190	haemophilia	3	187955	0.0015961
191	non hodgkin lymphoma	3	189352	0.0015844
192	gastroenteritis	3	204117	0.0014697
193	ergotism	2	177694	0.0011255
194	lumbago	2	178491	0.0011205
195	sprue	2	180431	0.0011085
196	vaginitis	2	185367	0.0010789
197	hidrosis	0	177074	0
198	haemoglobinopathy	0	177074	0
199	toxic shock syndrome	0	177074	0
200	fibrositis	0	177074	0
201	labyrinthitis	0	177074	0
202	hepatitis a	0	177074	0
203	pyorrhoea	0	177074	0
204	dropsy	0	177074	0
